# Review of Recent Nuclear Magnetic Resonance Studies of Ion Transport in Polymer Electrolytes

**DOI:** 10.3390/membranes8040120

**Published:** 2018-11-30

**Authors:** Stephen Munoz, Steven Greenbaum

**Affiliations:** 1 Department of Physics & Astronomy, Hunter College of the City University of New York, New York, NY 10065, USA; mehkie@gmail.com; 2 Doctoral Program in Physics, CUNY Graduate Center, New York, NY 10016, USA

**Keywords:** polymer electrolytes, ion transport, nuclear magnetic resonance (NMR)

## Abstract

Current and future demands for increasing the energy density of batteries without sacrificing safety has led to intensive worldwide research on all solid state Li-based batteries. Given the physical limitations on inorganic ceramic or glassy solid electrolytes, development of polymer electrolytes continues to be a high priority. This brief review covers several recent alternative approaches to polymer electrolytes based solely on poly(ethylene oxide) (PEO) and the use of nuclear magnetic resonance (NMR) to elucidate structure and ion transport properties in these materials.

## 1. Introduction

There is an ongoing quest to exploit the full potential energy embodied in the metallic Li^+^/Li electrochemical couple in practical and safe battery systems. Using a pure lithium anode material will increase volumetric and mass specific energy density by up to a factor of two while reducing battery cell manufacturing complexity—both key next steps for electrified transportation and consumer electronics [1]. To this end, solid state electrolyte materials have been under investigation for many decades, and the history of polymer-based systems has been with us since the 1970’s when polyethylene oxide (PEO) containing alkali metal salts was discovered to be an ionic conductor [2]. Though used for niche applications in thin-film all-solid-state configurations, ceramic or glassy solid electrolytes have recently experienced a strong resurgence in activity, due in part to the discovery of LGPS (Li_10_GeP_2_S_12_) [3] and its Si analogue [4]. Recently, a novel class of glassy electrolytes and electrode reactions has been proposed that work with both lithium and sodium ions [5], though these have been demonstrated in cells with operational potentials under 3 V. The reactive RF (Radio Frequency) sputter deposited LiPON (Lithium Phosphorous OxyNitride) system was demonstrated 20 years ago [6], and has proven to be difficult and costly to scale commercially, even when the useable cell area is on the order of square centimeters or smaller; large area cells are extremely problematic due to the formation pinholes and defects during deposition. Other variations of inorganic electrolyte systems have either been unable to suppress Li dendrites due to Li growth around ceramic grain boundaries have had very low room temperature conductivities, or have been unstable/not demonstrated with high potential cathode systems [7]. For large format applications where uniform thickness and composition over a wide geometric area are of paramount importance, ceramics and glasses electrolyte layers will pose substantial challenge as they suffer from structural rigidity resulting in loss of contact upon repeated cycling.

Although polymers can circumvent some of these issues, there are multiple critical performance parameters that dictate how a solid polymer will function in a battery environment, including ionic transport, mechanical stability, electrochemical stability (at high and low voltage) interfacial integrity, and ability to function at high rate and aerial capacity. Variants of the polyethylene oxide (PEO)-based polymer electrolytes have dominated the academic literature in this field. Approximately 40 years of research on PEO-based polymer electrolytes [8,9,10,11,12,13,14,15,16,17,18] has shown that achieving sufficiently high cationic conductivity (~10^−4^ S·cm^−2^ or better) at room temperature with high voltage cathode materials remains elusive. In the PEO system, the primary conduction mechanism involves the cooperative motion of cations and their coordinating polyether segments, which occurs in the amorphous phase of these often heterogeneous polymer-salt complexes above their glass transition temperature [10]. This has led to decades of effort on suppressing the crystalline phase and lowering the amorphous phase T_g_ for ambient temperature operation, with only incremental improvement in performance. Angell [19] defined a useful concept, the so-called decoupling index, which parameterizes the degree to which ionic and host structural relaxations are decoupled, and more recently Sokolov [20] recognized that solvent-free polymer electrolytes will probably never achieve a high enough level of conductivity unless the need for this coupling mechanism is eliminated or severely limited. Another consequence of the reliance on polymer segmental motion for ionic conductivity is that the Li^+^ transference numbers in PEO-based materials tend to be rather low, typically 0.25 or less [13].

Other necessary properties include: stability against lithium metal, ability to fill the material with a high volume percentage of active inorganic materials, swelling and solvent resistance, low electronic conductivity, and ease of processing.

To date, there have been few demonstrated practical device-level results showing the performance and stability of dry polymer electrolytes in functional energy storage devices using a lithium metal anode layer. Many published results elucidate conductivity as a function of temperature, and some assess the chemical stability of the material under anodic and cathodic potentials, though do not include data from full electrochemical cells including rate capability and cycle life studies. There are examples that show the cycling behavior of full cells, though these all have similar characteristics, including at least several of the following: cathode active material areal loading values are significantly lower than those used in practical lithium based batteries, cathode materials having a redox potential below 3.5 V, elevated temperature testing, and very small coin cell or swagelock test format, and all have current densities at 125 mA/cm^2^ and below). Table 1 is an accounting some of these results, many of which were recently published in premiere journals.

In reviewing the table, several things become apparent: none of these show performance at current densities (>0.5 mA/cm^2^) which are needed for practical devices, most are at high temperatures, and none stably incorporate cathode materials of practical importance with technologically relevant loading levels. Nonetheless, the current status of the inorganic sulfide solid electrolytes and the daunting scale-up problems they face, continues to motivate worldwide research into polymer electrolytes.

Ion transport characteristics remain a limiting factor on the practical applicability of many next-generation candidate battery electrolytes. NMR is especially well-suited to studying these properties, as it can easily probe much of the relevant time and length scales, while individually measuring the movement of the various constituents.

The net magnetization of the particles excited in the course of NMR experiments returns to equilibrium according to two relaxation profiles (longitudinal, or T_1_, and in-plane, or T_2_). Both the value of the relaxation rates and their characters (one- or multi-component) can be determined for those electrolyte components which can be tracked with an NMR-active nucleus. These relaxation rates are determined by intra- and inter-molecular spin interactions, and thus provide insight on the short-range dynamics of the system [33]. In fact, relaxation measurements have been used to probe dynamics since nearly the advent of NMR itself [34].

Long-range dynamics are also suitable for study by using pulsed field gradient NMR methods, which have been in use for the past five decades [35]. As the Larmor frequency of precession is determined by the local magnetic field strength, a magnetic field gradient encodes the position of particles in their phase. Pulsed magnetic field gradients make it possible to encode and decode positions while retaining high signal resolution. The resulting signal intensities can be compared to measure the self-diffusion coefficients, which can be used to calculate several properties germane to battery application.

This is all in addition to the structural characterizations which NMR is well-known for. The ability of NMR to investigate the coordination and solvation of particles in not only liquids, but also solids through the use of magic angle spinning, cross-polarization, and decoupling techniques has been leveraged for many years. More detailed explanations of their application can be found in the literature [36].

The purpose of this review is to examine several recent developments in the literature related to NMR-based investigations of ion transport in selected families of polymer electrolytes, most involving some modification of PEO. Though not exhaustive, we believe that the examples we have chosen to highlight are representative of the majority of current approaches to viable polymer electrolytes, with NMR as a primary analytical tool.

## 2. PEO and Ceramic Composite Electrolytes

Poly(ethylene oxide) was among the first polymers to be discovered to be an ionic conductor, and the decades since this discovery has seen much time and energy put into reaching its potential as a solid polymer electrolyte. It forms the basis of many more complex polymer systems, including many composites and copolymers [12,37], thanks to the wealth of information on its mechanical and electrochemical properties. Although PEO tends to suffer from low room-temperature conductivity values, the significant advantages it brings in terms of promoting Li salt dissolution, as well as its mechanical properties as a solid polymer, justifies the continued interest in its refinement as an electrolyte.

Polymer/ceramic composite electrolytes are an attractive option for customized mechanical and electrochemical properties. For decades, it has been known that incorporating certain ceramic materials into the polymer matrix can improve the ionic conductivity of the material, mitigating one of the key weaknesses of solid polymer electrolytes [38,39]. This effect is achieved via surface groups of the ceramic particles modifying local structure, as suggested by studies investigating particles of reduced size [40,41]. The particles are believed to affect the recrystallization of the polymer chains, resulting in amorphous regions conducive to fast Li^+^ ion transport [42]. Inclusion of nanowires in lieu of particles can provide a long-range network for improved lithium mobility [43,44]. Ceramic additives enhance the ability of the electrolyte to form a stable interface with electrodes [39,45]. Certain combinations of polymer and ceramic can even result in a greatly increased Li^+^ ion transference number due to cross-linking of the polymer chain promoted by the presence of the ceramic filler, resulting in Li^+^-preferred transport channels near the particles [21].

More recently, “polymer-in-ceramic” electrolytes composites have demonstrated good mechanical properties, high discharge capacities, and good capacity retention in solid state lithium-metal batteries [46]. Incorporation of Li-ion conductive ceramics with a high shear modulus can have the effect of increasing the mechanical resistance to lithium dendrite formation. This, paired with the somewhat Li-insulating nature of the polymer matrix, results in the suppression of dendritic growth while still allowing proper conduction of lithium ions [47]. Thanks to this improved interfacial stability, lithium-metal compatible ceramic composite electrolytes have shown promising behavior [48,49].

A recent study by Zheng et al. [50] focused on elucidating the somewhat complicated nature of Li-ion transport through composite materials, where the ions might transport through the polymer matrix, through the ceramic fillers, and/or through their interfaces. Cubic-Li_7_La_3_Zr_2_O_12_ (LLZO) dry powder was added to a polymer matrix consisting of poly(ethylene oxide) and lithium bis(trifluoromethanesulfonyl)imide (LiTFSI), then ball milled. The resulting slurry was solution cast and dried into a composite film. Several films were cast with different wt. % fractions of LLZO, from 5 wt. % to 50 wt. %. Finally, a separate sample was cast with tetraethylene glycol dimethyl ether (TEGDME) included, at 20 wt. % TEGDME and 50 wt. % LLZO.

^6^Li solid-state magic-angle spinning NMR was performed to characterize the local structure and dynamics of the lithium ions. The results contain a peak representative of LLZO decomposed through the ball-milling process at 1.3 ppm (relative to LiCl). The results also show a new peak at 1.8 ppm relative to LiCl, indicative of the LLZO–PEO interface [51]. In the sample containing TEGDME, this peak was observed to slightly shift and its area integral to increase. This, along with the increased intensity of the decomposed LLZO peak, confirmed that the TEGDME assists in the breakdown of LLZO, and may play a role in converting more of it to an interfacial complex.

Broadening suggestive of disorder of the local environments for lithium ions is observed in the LiTFSI peak at higher concentrations of LLZO, characteristic of reduced polymer crystallization. A slight reduction in the FWHM in the sample containing TEGDME can be attributed to a partial averaging of the anisotropic interactions due to the increased mobility of the lithium ions. Evidence of this increased mobility was also present in a reduced T_1_ of the decomposed LLZO signal for the sample containing TEGDME.

**Figure 1:**
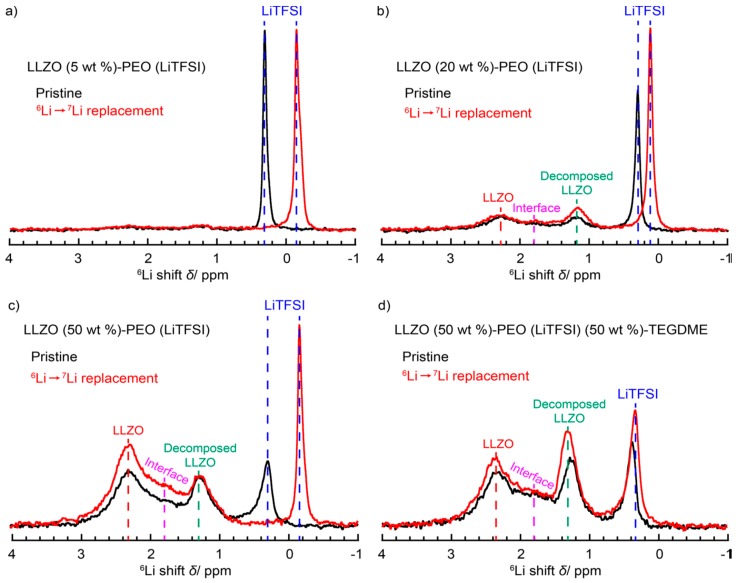
^6^Li NMR comparison of pristine and cycled LLZO (5 wt. %)-PEO (LiTFSI), LLZO (20 wt. %)-PEO (LiTFSI), LLZO (50 wt. %)-PEO (LiTFSI), and LLZO (50 wt. %)-PEO (LiTFSI) (50 wt. %)-TEGDME [50]. Reprinted with permission from [50]. Copyright 2018 American Chemical Society.

Li-ion transport was further investigated by using ^6^Li metal electrodes in symmetric cells, and then cycling them to enrich the ^6^Li in the polymer electrolyte via isotopic exchange. The low natural abundance of the ^6^Li isotope (7.6%) means that the pathways preferred by Li-ion transport should experience a noticeable enrichment of ^6^Li (Figure 1).

The results reveal that for the 5 wt. % LLZO sample, an enrichment in the LiTFSI signal is observed, along with a shift in the peak resonance. This change in the ions’ electronic environment is consistent with reduced PEO-Li interaction in amorphous phase PEO, leading to faster Li-ion conduction (Figure 1a).

Combined with the T_1_ data and CPMAS (^1^H–^6^Li) showing very little interaction between LLZO and the PEO matrix, the authors were abler to conclude that the 20% LLZO composite still mainly conducts lithium via the polymer matrix, with the decomposed LLZO assisting the ionic conduction.

The LLZO 50 wt. % sample produced spectra suggesting that the main conduction pathway had changed, with the bulk of enrichment occurring in the LLZO peak, with some in the LiTFSI and interface peaks (Figure 1c). No enrichment was observed to occur in the decomposed LLZO peak. There was now enough LLZO to form a coherent network for the ions to travel through.

Finally, the LLZO 50 wt. % + TEGDME sample spectra revealed that the Li-ion conduction pathway changed again, back to the decomposed LLZO and LiTFSI. This is consistent with TEGDME’s high natural ionic conductivity, as well as its ability to reduce PEO crystallization, resulting in preferred movement for lithium through the polymer/TEGDME matrix.

Further electrochemical measurements via Electrochemical Impedance Spectroscopy (EIS) would reveal that the 50 wt. % LLZO sample demonstrates the lowest conductivity of the samples (<1 × 10^−5^ S/cm), due to the PEO pathways being blocked and the LLZO network providing poor conductivity on its own. In contrast, the sample containing TEGDME demonstrated a much higher conductivity (>5 × 10^−5^ S/cm) due to the TEGDME’s ability to facilitate ion conduction channels through the PEO. In fact it can be argued that due to these interfacial issues, there is limited advantage to incorporating a highly conducting ceramic over a non-conducting (in the bulk phase) one [7,12,14,21].

Another study by Lago et al. [23] leveraged solid-state NMR to study a plasticized PEO-based Solid Polymer Electrolytes (SPE) containing anions grafted onto ceramic nanoparticles. The idea was to combine the improved conductivity and electrochemical stability of a lithium-only conduction polymer with the increased ionic dissociation, inhibited crystallization, and improved mechanical properties associated with incorporated ceramic nanofillers [45].

Variable-temperature ^19^F solid-state NMR was performed on two samples: one, the classic PEO(LiTFSI) [EO:Li 20:1], and one composite sample comprised of 5 nm Al_2_O_3_ ceramic nanoparticles functionalized simultaneously with lithium 4-[2-(trimethoxysilyl)ethyl]benzene-1-sulfonyl [(trifluoromethyl)-sulfonyl]amide and PEG9 trimethoxysilane [EO:Li 50:1] in PEO:PEGDME 1:1 (the choice of nanoparticles was based on a previous work) [52].

**Figure 2:**
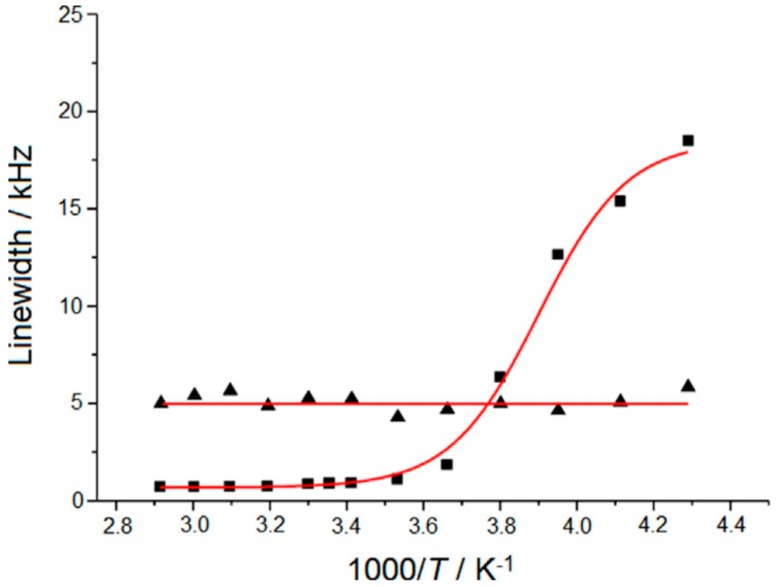
Comparison of ^19^F NMR linewidths for (triangle) Al_2_O_3_-PEG9-anion (5 nm). [EO]/[Li]~50 in PEO/PEGDME~1, and (square) LiTFSI in PEO [EO]/[Li]~20 as a function of temperature [23] © 2015 Wiley-VCH Verlag GmbH & Co. KGaA, Weinheim.

Figure 2 shows the comparison of the resultant linewidths of the anion signals in the two samples as a function of inverse temperature. A clear difference in the linewidth response is evident. The linewidth in the LiTFSI−PEO system is heavily dependent on temperature, a consequence of the fact that the mobility of the fluorine in the LiTFSI molecules is coupled to the mobility of the PEO matrix. As the temperature increases, the PEO segments become much less rigid, allowing greatly increased freedom of movement to the LiTFSI molecules, whose molecular tumbling averages out the local anisotropic interactions and results in a much-narrowed NMR peak. To the contrary, the relative temperature-independence of the sample containing functionalized Al_2_O_3_ nanoparticles indicates that the local mobility of the anions is decoupled from that of the polymer matrix. Furthermore, the larger linewidths in the sample containing nanoparticles at higher temperatures confirms that, although their movement is decoupled from that of the polymer, it does experience restriction due to its association with the Al_2_O_3_ nanoparticles.

EIS measurements would reveal that this composite material shows conductivity approaching 10^−4^ S/cm at 70 °C. This electrolyte was then used to create an Li-metal/LiFePO_4_ cell which demonstrated better cycling performance than previous Li-metal batteries with composite polymer electrolytes [53]. This, combined with the respectable conductivity and high cation transference resulting from the immobilization of anions, means that this approach could represent a viable path forward on the development of a practical solid-state battery.

These results demonstrate the ability of solid-state NMR to discern the different contributors to ion transport in these complex materials. Polymer/ceramic composites represent a polymer electrolyte family with excellent potential, thanks to its mechanical and electrochemical customizability and compatibility. NMR can be instrumental in developing models to guide future design of these promising electrolyte candidates, or in verifying critical aspects of their performance.

## 3. Copolymers, Block Copolymers, and Polymer Blends

There is significant interest in the use of copolymers as battery electrolytes, due to the fact that different components can be used to selectively engineer the nanostructure, theoretically leading to advantageous macroscale properties [54,55]. Crystallization of polymer-based electrolytes has been shown to limit the conductivity below practical application levels [56], but incorporating copolymers has been shown to be a viable way to mitigate this crystallinity [57,58,59]. Phase separation can assist in both improving conductivity and in inhibiting lithium dendrite growth through mechanical rigidity.

**Figure 3:**
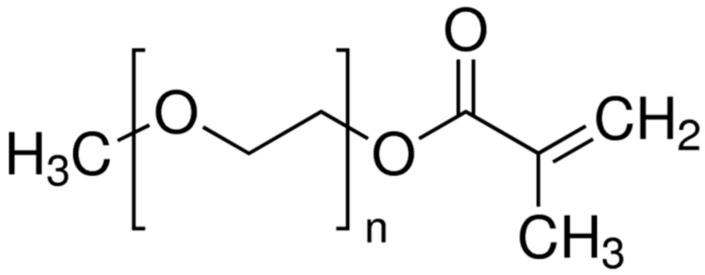
Poly(ethylene glycol) methyl ether methacrylate (PEGMA) chemical structure.

A recent study by Daigle et al. investigated the Li^+^ ion mobility in comb-like copolymers via solid-state NMR [60]. These comb-like polymers were based on poly(styrene) (PS) backbone fashioned through anionic polymerization. The purpose of this backbone was to provide mechanical reinforcement to inhibit lithium dendrite growth via the phenyl groups. Poly(ethylene glycol) methyl ether methacrylate (PEGMA, shown in Figure 3) was grafted to assist in Li^+^ conductivity by suppressing crystallinity. LiTFSI salt was incorporated to provide charge carriers. Several samples were created with differing ratios of PEGMA to PS (2.6:1, 3.9:1, and 30:1). Solid-state cross-polarization (CP) and direct acquisition ^13^C NMR measurements were performed to characterize the structure of the polymers, while ^7^Li NMR measurements were performed to track the li-ion transport mechanics.

The structural analysis revealed that, as expected, the PEGMA backbones were more rigid than the pendant groups. However, a signal attributed to the pendant groups was acquired in the CP measurements; due to the fact that some rigidity is necessary to facilitate the magnetization transfer necessary for a CP measurement, the authors concluded that the coordination between the pendant groups and lithium salts resulted in this rigidity.

^7^Li NMR was then used to elucidate the Li-ion transport mechanisms. The samples with the lower ratios of PEGMA to PS displayed conductivities above 10^−4^ S/cm at 60 °C. This approaches what could be considered high enough conductivity for practical application. This suggests the potential for these materials for use in energy storage, and the importance of understanding the mechanisms underlying their operation.

Lithium ion diffusion was deduced by examining the linewidths of the peaks produced in the spectra (it is possible to relate these linewidths to transverse relaxation of the signal, mediated by short-range interactions). Similarly, short-range motion can be correlated with longitudinal relaxation times, also measurable via NMR.

The linewidths, measured across the three samples as a function of temperature, reveal that the lithium signals produce very sharp peaks when compared to copolymers based on polyurethane-poly(dimethylsiloxane) [61]. This is correlated with more mobility of the ions, which is corroborated by the fact that conductivity (measured here by AC impedance spectroscopy) is several times higher than in that previous material, in the case of the samples with lower PEGMA/PS ratio. In addition, when ^1^H decoupling was applied, no change was observed in the signal, leading the authors to conclude that the lithium-ion mobility was high enough to motionally average out ^1^H–^7^Li dipolar interactions.

**Figure 4:**
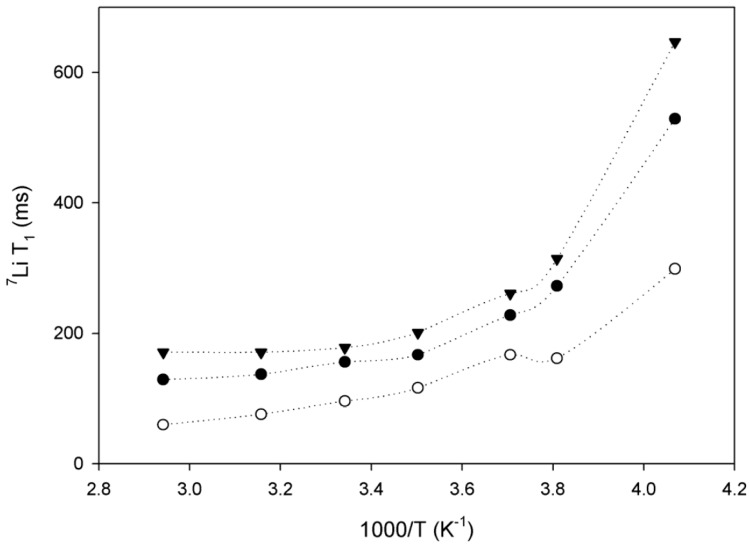
Evolution of ^7^Li linewidth as a function of inverse temperature for sample 1 (**black triangles**), sample 2 (**open circles**) and sample 3 (**black circles**) [60] (OPEN ACCESS).

T_1_ as a function of temperature, shown in Figure 4, would reveal that sample 2 (PEGMA:PS 3.9:1) demonstrates the highest lithium mobility, while at the same time showing a weaker temperature dependence than the other two samples. However, all three samples demonstrate a sharp drop in mobility around 263 K.

The similarity between the lithium ion mobility and PEGMA chain mobility allowed the authors to conclude that their movement is correlated.

Another recent study reported on eight PEO-polycarbonates [62]. This study was motivated by research showing that aliphatic polycarbonates could enable room-temperature cycling [63,64]. The authors prepared several different samples of PEO-PC polymer, varying both the ratio of PEO to PC and the LiTFSI salt concentration. ^1^H, ^13^C, ^19^F, and ^7^Li NMR experiments were performed to characterize the structure and local dynamics of the system.

**Figure 5:**
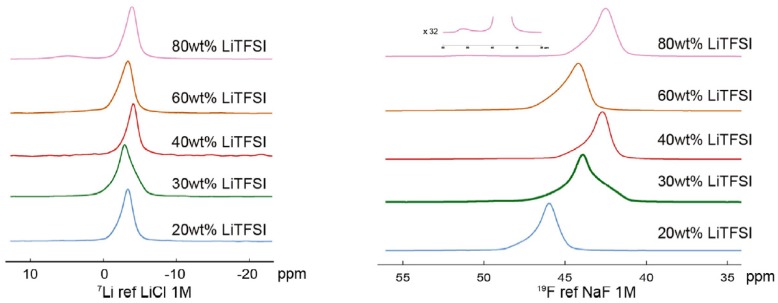
(**left**) ^7^Li NMR spectrum of PEO34-PC at 343.15 K of different wt. % LiTFSI samples. (**right**) ^19^F NMR spectrum of PEO34-PC at 343.15 K of different wt. % LiTFSI samples [62] Reprinted from [62]. Copyright 2018, with permission from Elsevier.

The authors elected to investigate the effect of varying salt concentration on the PEO-PC (34:1) sample, owing to it displaying the highest room temperature conductivity as measured by AC impedance spectroscopy across the entire temperature range measured. ^7^Li relaxation experiments would reveal that a new signal appears for both the ^7^Li and ^19^F spectra at the highest concentration of salt, 80 wt. % LiTFSI (despite significant shimming issues affecting the lineshapes, the authors note a discernable difference between the attenuation of the two peaks, concluding that the secondary peak is not an artifact of shimming. However, it should be noted that the very broad lineshapes of the secondary peaks can affect the accuracy of any relaxation times derived thereof.) The authors ascribe this secondary peak to the formation of LiTFSI aggregates. The spectra are displayed in Figure 5.

The BPP (Bloembergen, Purcell, and Pound) model [34] was fitted with the resulting T_1_ measurements to calculate the correlation time, activation energy, and quadrupolar coupling constant for the samples of varying salt concentration. The activation energy was found to drop dramatically with higher salt concentrations, consistent with faster reorientational dynamics. Higher correlation times were calculated for the ion aggregates, consistent with slower dynamics and less mobility.

^7^Li and ^19^F pulsed field gradient NMR was also performed on the PEO-PC (34:1) samples with differing salt concentration. Of note is a sharp uptick in the diffusion coefficients of both ^7^Li and ^19^F at 80% wt. LiTFSI, which the authors note contradicts the AC impedance spectroscopy-measured conductivity values which follow a consistent downward trend with higher salt concentration. This is explained through the fact that in many systems, the diffusion can be so slow, or the relaxation so fast, that certain species may go undetected in the course of the experiment. It is very likely that the Li ion’s share of the lithium signal is dying out before ever being acquired by the spectrometer, resulting in diffusion coefficients being calculated from the attenuation of just a tiny fraction of the “true” signal. This is an illustration of the importance of tempering conclusions made from solely NMR by comparing against other methods of measurement.

Another recent study, carried out by Timachova et al. [65], focused on a nanostructured block copolymer electrolyte. These electrolytes are of interest because they can form nanoscaled ordered regions of alternating phase, which enables the kind of combinations of rigidity and conductivity necessary in a practical battery electrolyte. Much work has focused on the characterization of these materials due to their attractiveness as electrolytes [66,67]. In 2016, Chintapalli et al. [68] reported a study of polystyrene block poly(ethylene oxide) (SEO) mixed with LiTFSI. Through a combination of differential scanning calorimetry (DSC) and AC impedance spectroscopy, they determined that the maximum conductivity of the SEO occurred at very different salt concentrations than in the PEO (*r* = 0.21 as opposed to *r* = 0.11). This is due to inhibited grain growth, which increases the ionic conductivity of the block copolymer.

Timachova et al. applied PFG-NMR to study a polystyrene-b-poly (ethylene oxide) copolymer/lithium bis(trifluoromethanesulfonyl)imide solid electrolyte as a function of salt concentration. Their goal was to characterize the local anisotropic nature of Li-ion diffusion due to the lamellar layers, and to obtain the isotropic continuum transport properties (a first for block copolymer electrolytes).

The SEO in the samples was synthesized via sequential anionic polymerization of styrene followed by ethylene oxide [69]. Electrolytes of several different LiTFSI concentrations were then prepared (*r* = [Li]/[EO] = 0.03, 0.06, 0.12, 0.18, 0.24, and 0.3).

**Figure 6:**
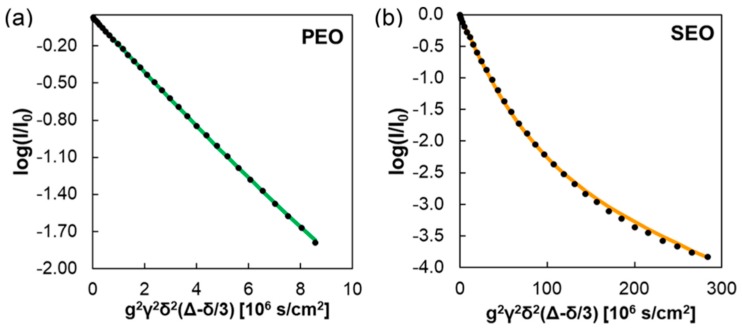
PFG-NMR signal attenuation of ^19^F seen in (**a**) PEO(5)/LiTFSI at *r* = 0.06 and (**b**) SEO(16−16)/LiTFSI at *r* = 0.18 [65]. Reprinted with permission from [65]. Copyright 2018 American Chemical Society.

Pulsed-field gradient NMR was performed on the electrolyte samples, targeting both ^7^Li and ^19^F nuclei to track the movement of cations and anions. Initial comparison of the attenuation curves of a traditional PEO electrolyte with that of the SEO block copolymer reveals a clear difference, as seen in Figure 6.

A linear relationship between the normalized signal intensity and the square of the gradient strength is indicative of isotropic diffusion in this case, as illustrated by the PEO result. In contrast, the SEO electrolyte produced a nonlinear relationship, indicating anisotropic diffusion. The curve through Figure 6b represents the best fit of the anisotropic diffusion coefficient with D‖ (diffusion along the lamellae) and D⊥ (diffusion perpendicular to the lamellae).

**Figure 7:**
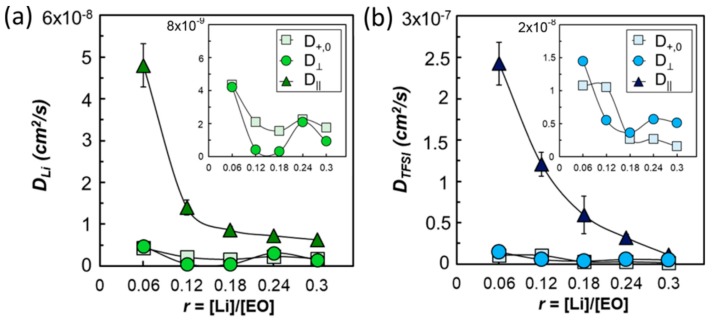
Parallel, D‖, and perpendicular, D⊥, diffusion coefficients and the Stefan−Maxwell diffusivities of (**a**) Li and (**b**) TFSI in SEO(16−16) as a function of salt concentration, *r*, at 90 °C [65]. Reprinted with permission from [65]. Copyright 2018 American Chemical Society.

Figure 7 shows the resulting values through the PEO-rich lamellae (associated with D‖) and across the PEO/poly(styrene) boundaries (associated with D⊥). AC impedance spectroscopy was performed to measure the conductivity, and steady-state current and restricted diffusion measurements were performed to help calculate the steady-state transference number. These values were then used to calculate the Stefan-Maxwell diffusion coefficients, represented by the light squares in the plots. The calculated diffusion coefficients representing the Li-PEO and TFSI−PEO interactions are consistent with the D⊥ values measured by PFG-NMR. This strong agreement from two different measurements allowed the authors to conclude that the electrochemical performance is strongly coupled with ion transport through defects in this system.

**Figure 8:**
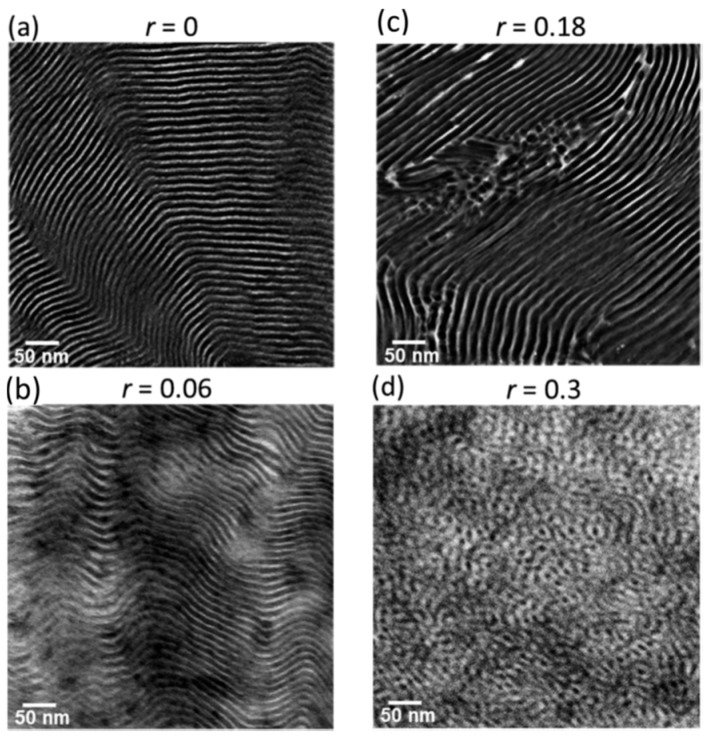
Dark-field transmission electron microscopy images of SEO(16−16) at (**a**) *r* = 0 and (**c**) *r* = 0.18, reproduced from [68] and at (**b**) *r* = 0.06 and (**d**) *r* = 0.3 measured in this work. The bright phase is poly(ethylene oxide) [65]. Reprinted with permission from [65]. Copyright 2018 American Chemical Society.

This result is significant because it represents the first time that the local anisotropic nature of diffusion has been characterized in a block copolymer electrolyte. The authors would leverage this new information to establish an NMR-based “morphology factor” representing the degree of isotropy of diffusion. They would find that this factor indicates low isotropy at low concentrations of salt (close to that expected for an ideal lamellar system with D⊥ = 0), and high isotropy with higher concentrations of salt. Transmission electron microscopy (Figure 8) would confirm that the lamellar structure contains significantly more defects with higher salt concentration. This provides further evidence that the NMR measurements were able to accurately decouple the in-plane and through-plane diffusion values. The results indicate that transport in the bulk is strongly influenced by defects in the structure, providing guidance for further optimization of these materials.

**Figure 9:**
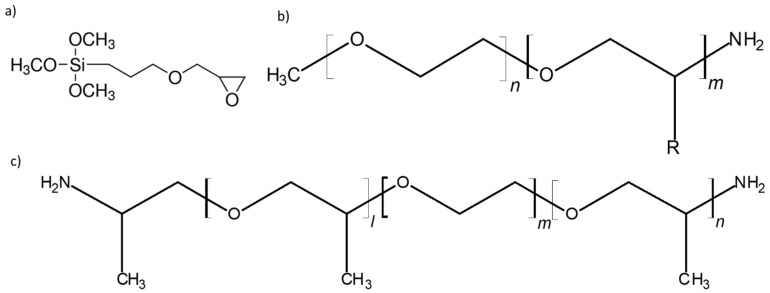
Structures of (**a**) (3-glycidyloxypropyl)trimethoxysilane (GLYMO), (**b**) Jeffamine M-2070, and (**c**) Jeffamine ED2003.

Another study by Liu et al., in 2017 [70], would focus on a blended hybrid solid polymer electrolyte. Blended polymers are attractive due to the ease of synthesis while still providing a high degree of control over the mechanical properties of the end product [71,72,73]. This particular material studied was created by blending two organic-inorganic hybrids. One hybrid consisted of (3-glycidyloxypropyl)trimethoxysilane (GLYMO), an organosilane, cross-linked with a monoamine-based polyether (Jeffamine M-2070); the second consisted of GLYMO and poly(ethylene glycol) diglycidyl ether (PEGDGE) reacted with a diamine-based polyether (Jeffamine ED2003). These structures are illustrated in Figure 9. These hybrids would be combined in different ratios and LiClO_4_ salt added to create several electrolyte samples for examination. Solid-state NMR was performed on ^13^C, ^29^Si, and ^7^Li nuclei to characterize the lithium-ion mobility and confirm the structure of the material.

AC impedance measurements would suggest that the ion transport was linked to the segmental motion of the polymer matrix, as is common in many solid polymer electrolytes. The authors determined that a peak ionic conductivity should occur at a salt concentration of [O]/[Li] = 16. This was based on the fact that increasing the salt concentration can have competing effects on conductivity—higher concentration increases the amount of charge carriers, but too high of a concentration can lead to ion aggregation and impede mobility. It should be noted here that some recent studies have shown that certain salt concentrations outside the assumed window of interest can yield competitive performance via interionic interactions [74].

^7^Li static linewidths were measured across a range of temperatures from −90 °C to 90 °C. Measurements carried out without proton decoupling would be characterized by a plateau of broad linewidths ~5 kHz at temperatures below −60 °C, and another flat region of narrow linewidths above 60 °C. Activation energies were calculated from these results to be 0.15 eV for the sample synthesized from hybrid 1:hybrid 2 (70:30) with [O]/[Li] = 32 (denoted in the article as MP(70:30)-32 and 0.14 eV for MP(70:30)-16, which is comparable to that of similar systems in the literature [75]. With proton decoupling applied, a sharp decrease in the linewidth at lower temperatures was observed, leading the authors to conclude that about 80% of the interaction causing broadening was due to ^7^Li–^1^H dipolar interactions. VTF fitting of conductivity measured by AC impedance spectroscopy would confirm that the ionic conductivity is strongly coupled to the polymer segmental chain motion.

Following up on this, ^1^H-decoupled ^7^Li MAS measurements were performed in the same temperature range. These measurements revealed that the coordination between Li-ions and the ether oxygens in the polyethers in M-2070, ED2003, and PEGDGE produced the strongest signal, while that of the ^7^Li coordinated with GLYMO oxygen was extremely difficult to detect. The authors ascribe this lack of signal to low GLYMO concentration in the sample.

Although the MP(70:30)-16 sample showed the highest room temperature conductivity value at over 1x10^-4^ S/cm and an electrochemical stability window approaching 5 V, test cells incorporating it showed significant irreversible capacity loss upon cycling due to the suspected formation of a passivation layer. However, examination after cycling would show that the membrane showed no signs of mechanical decomposition or particle aggregation.

## 4. Crystalline Polymer Electrolytes

Acceptable conductivity cannot occur in crystalline polymer electrolytes unless the ionic motion can be effectively decoupled from the polymer matrix [76]. In fact, this decoupling is believed to be an important step toward developing polymer electrolytes of any type that can provide the ionic conductivities necessary for practical application [20]. In 2001, Gadjournova et al. showed how crystallinity can be a boon for cation transport, due to the regularity of ordered diffusion pathways [76]. A number of approaches have been suggested in recent years for enhancing the conductivity, including anionic doping, replacement of the ends of the polymer chains with glymes to enhance disorder [77,78,79], and stretching of the polymer to align the chains and enhance transport along the longitudinal axis [80,81].

Recently, Yan et al. reported a new crystalline solid polymer electrolyte consisting of a PEO-urea-LiTFSI complex [82]. Their investigation was motivated by a previous study on a similar ternary structure involving α-cyclodextrin, which demonstrated fast Li-ion movement through the resultant structure. Urea was chosen as the next candidate, thanks to the formation of a crystalline inclusion compound in PEO-urea binaries [83,84,85].

The ternary complex was prepared by dissolution of PEO, urea, and LiTFSI in acetonitrile (with varying concentrations of the LiTFSI salt), followed by stirring, casting at 40 °C, and drying. Wide angle x-ray spectroscopy was used to verify the crystalline nature of the resulting compounds. The α-PEO-urea-LiTFSI complex forms the same crystalline structure as α-PEO-urea, only slightly more compact. A high level of crystallinity is maintained. The highest-conductivity samples were investigated via solid-state ^1^H–^7^Li and ^1^H–^19^F cross-polarization MAS NMR to determine Li^+^ and TFSI^−^ ion coordination.

These tests would show evidence of correlation between Li^+^ ions and both NH_2_ (at ~6 ppm) in urea and CH_2_ (at ~4 ppm) in PEO. This suggests that Li^+^ is present in the crystalline inclusion structure, where both the urea and PEO are known to be present. The same trend is noted for the ^1^H–^19^F tests; TFSI^−^ anions are correlated with both the urea and PEO, suggesting their presence in the hexagonal urea channel as well. Of note here is the presence of two peaks in the ^19^F spectrum, which the authors ascribe to differing environments. From an NMR perspective, relaxometry studies, as well as diffusometry, could be of use here in further elucidating differences between the environments (however, specialized systems would be required given the relatively slow diffusion expected from their reported conductivities).

The authors describe the resultant system as consisting of the aforementioned inclusion structure consisting of urea channels containing PEO chains and Li^+^ and TFSI^−^ ions. There is evidence that urea promotes ionic dissociation between Li^+^ and TFSI^−^ [86]. In addition, the authors hypothesize that the channels may trap the larger TFSI^−^ anions, allowing the Li^+^ ions to travel freely. This would produce conductivity and transference numbers more favorable for battery operation.

Impedance spectroscopy was also used to determine the conductivities of the sample. The highest conductivity material demonstrates a conductivity of ~6 × 10^−5^ S/cm at 30 °C. This value compares favorably to previous highly crystalline polymer electrolytes, and even to some amorphous species, but it is still orders of magnitude below what would be required for a commercially viable battery. However, this study demonstrates that exploitation of inclusion complexes similar to these could facilitate competitive conductivities and transference numbers.

**Figure 10:**
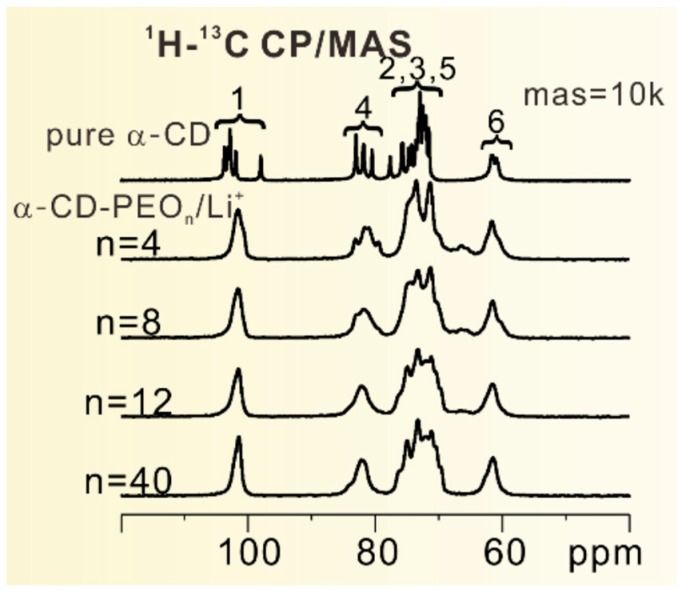
The disappearance of the signal splitting indicates the formation of α-CD-PEO inclusion complex [87] © 2018 WILEY-VCH Verlag GmbH & Co. KGaA, Weinheim.

In another study, Fu et al. [87] reported a crystalline polymer electrolyte based on self-assembled α-cyclodextrin (CD), polyethylene oxide (PEO), and Li^+^ salts. Through the literature, they identified two key goals to increasing the ionic conductivity: to decouple the Li^+^ ions from the polymer chain segments [24,88,89,90,91,92,93,94,95,96] and to generate long-range pathways for bulk ionic transport [97]. Along these lines, they combined their self-assembly approach to creating ordered nano-tunnels with alteration of the conformational sequence of PEO to inhibit interaction between Li^+^ ions and the polymer segments. Samples of differing α-cyclodextrin to PEO ratios were synthesized following previous works [98,99]. ^1^H–^13^C CP MAS NMR and wide angle X-ray diffraction measurements were performed to verify the tunnel structure of the PEO chains. The ^13^C NMR spectra are displayed in Figure 10.

The NMR results verify that the splitting is no longer resolved in the complex samples compared to the neat α-CD, which has been associated in the literature with the formation of inclusion complexes [100,101,102].

**Figure 11:**
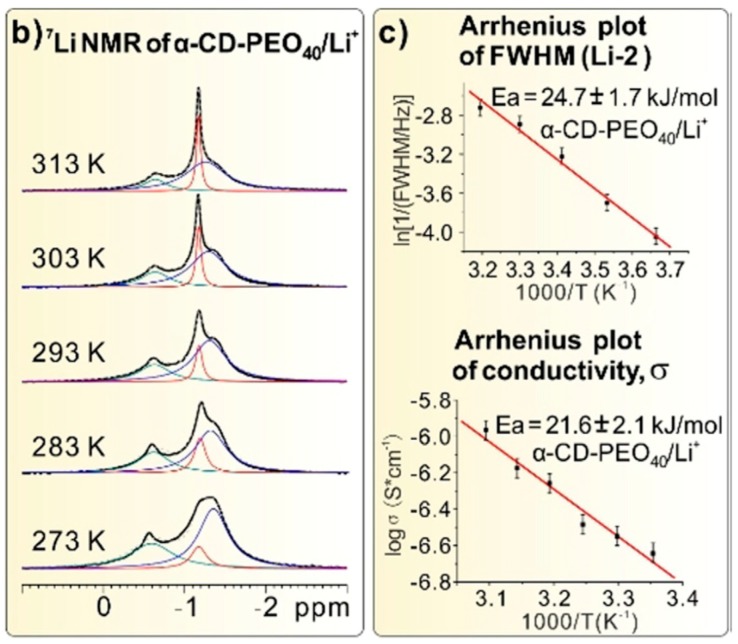
(**Left**) Temperature-dependent ^7^Li NMR spectra of α-CD-PEO40/Li^+^ from 273 K to 313 K. (**right**) Top: The Arrhenius plot of the line widths of Li-2 from the temperature-dependent ^7^Li NMR; Bottom: The Arrhenius plot of the conductivities of α-CD-PEO40/Li^+^ [87] © 2018 WILEY-VCH Verlag GmbH & Co. KGaA, Weinheim.

Static ^19^F NMR measurements would reveal that the lineshape of the signal changes very little over the temperature range 0–40 °C, with a broadness indicative of low mobility. In contrast, the ^7^Li static NMR measurement (spectra shown in left of Figure 11) would manifest a narrow signal associated with high mobility. This narrow signal at −1.15 ppm would be designated Li-2 by the authors, associated with Li^+^ ions between polymer chains and CDs. Broader signals at −1.31 ppm and −0.64 ppm would be assigned to Li^+^ ions strongly associated with the polymer chains and with the assembled CDs, respectively.

In addition, the narrow Li-2 signal broadened significantly with lower temperature. This indicates that the motion of the Li cations is decoupled from that of the anions, at least in the higher temperature regime. This has important implications for the applicability of this material, as ion pairing can significantly attenuate the practical conductivity of an electrolyte.

Based on the linewidth of Li-2 observed in the NMR measurements, plotted in right of Figure 11, the activation energy was calculated to be about 21.6 kJ/mol, which agrees, within error, with the activation energy calculated from the temperature dependence of the conductivity (also shown in right of Figure 11) measured by EIS (although the authors are careful to mention that this could be coincidental). This low value indicates that the motion of the Li^+^ ions is coupled much less strongly than in systems such as oxygen-based superionic conductors or EO/Li^+^ complex crystals [103,104]. This decoupling, in tandem with tunnel structure providing long-range Li^+^ transport pathways, produces a high EIS-measured conductivity on the order of 1 × 10^−3^ S/cm at room temperature in the sample with the lowest lithium concentration.

Further ^2^H solid state NMR measurements would provide evidence that the PEO chains were forming all-trans conformation sequences. Simulations show that no stable structure exists for Li^+^ ions to coordinate with the PEO chains in such a conformation sequence, further reinforcing the idea that the Li^+^ ions are very weakly coupled to the PEO chains in this system. In the PEO_4_ sample, the PEO chains are more likely to conform in the trans-trans-gauche sequence, resulting in stronger coupling between ions and the polymer matrix, with resultant lower ion mobility and conductivity. These results have important implications for the refinement of solid polymer electrolytes which facilitate fast Li^+^ ion transport, both through the engineering of nanostructure and through attenuation of the interaction between ions and the polymer.

## 5. Sodium-Conducting Electrolytes

Sodium chemistries represent an attractive alternative to the established lithium-based technology, due to similar properties and much higher abundance. They are limited by a lack of compatible electrodes, which are in turn limited by the need for a suitable electrolyte. A recent study by Pope et al. reports an investigation of a single-ion conducting Na electrolyte [105]. Single-ion conductors promote facile transport of one ion while trapping the counter ion. With proper engineering this can lead to extremely high transference numbers and result in optimized battery performance.

This particular study is motivated by previous works identifying poly(2-acrylamido-2-methyl-1-propane-sulfonate (PAMPS) as a suitable base for such an electrolyte, thanks to reduced cation tethering to the immobilized anions [106,107,108].

**Figure 12:**
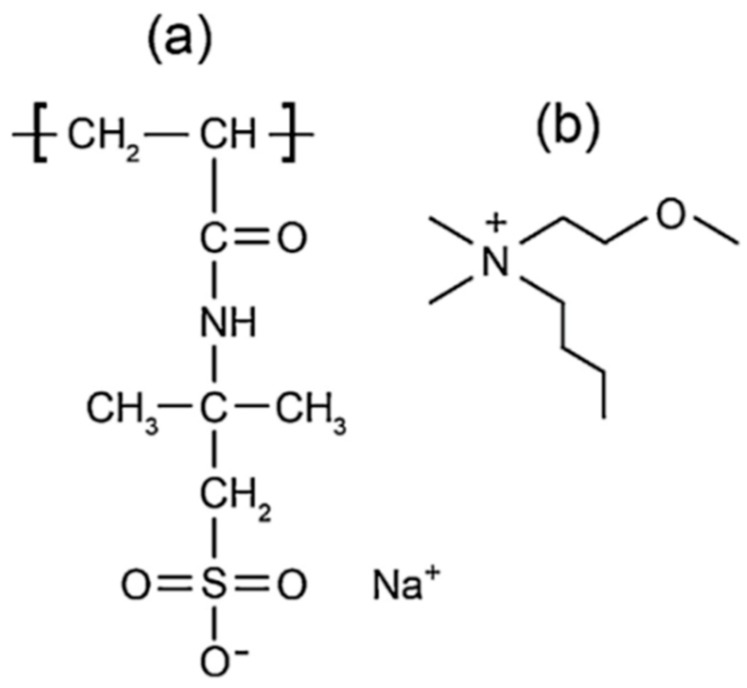
Structure of the ionomer. (**a**) The poly(2-acrylamido-2-methyl-1-propane-sulfonic acid) (PAMPS) monomer and (**b**) the dimethylbutylmethoxyethyl ammonium cation (N114(201)).

Further studies have shown that incorporation of a bulky IL quaternary ammonium cations can reduce the T_g_ by inhibiting crosslinking, leading to increased conductivity [109,110] and have indicated that ether group-containing additives can partially solvate Na^+^ ions, further assisting in their transport [111]. Putting these ideas together, the authors created an electrolyte by combining a PAMPS homopolymer with an ether group-functionalized quaternary ammonium ion as illustrated in Figure 12. They performed solid-state ^23^Na NMR experiments to further investigate the Na^+^ transport mechanism.

Two-component T_1_ relaxation results would show strong evidence of at least two different Na populations. The larger-linewidth component was assigned to a less-mobile population, and corresponded to a larger T_1_. The difference in linewidths implies an expected difference in T_2_ as well, making an exact population split calculation unfeasible due to the differing attenuation effects. Variable-temperature linewidth measurements would reveal that the relaxation times of the different populations become more and more similar above the glass-transition temperature. The authors concluded that the less mobile Na signal can be attributed to ions bound to anionic sulfate groups, with the more mobile signal attributed to unbound Na^+^ ions.

Despite the T_g_-lowering effect of the ammonium cations, the glass-transition temperature remains too high in this system for practical room-temperature application. Regardless, this study demonstrates the selective ability of NMR to decouple the mobilities of different species, even in multi-cationic systems.

Another recent study focuses on the use of electrospinning to fabricate a sodium-ion conducting PEO-based membrane [112]. Sodium is an attractive alternative to lithium-based chemistries, thanks to its abundance and cost advantages. Recent research has shown that electrospinning can produce membranes with enhanced performance when compared to traditional solution-cast membranes [113]. Samples were created by combining PEO with NaBF_4_ and succinonitrile (SN) in different ratios.

AC impedance spectroscopy would reveal that, near room temperature, the best conductivity was observed in the sample containing (PEO:SN:NaBF_4_) in the ratio (18:0:1)—indicating that the inclusion of the succinonitrile plasticizer did not provide an improvement in conductivity.

^19^F and ^23^Na solid-state NMR was then performed at ~265 K to investigate the local environments. The results would indicate significant mobility differences between the samples of different concentrations. In the case of the ^19^F spectra, a very broad linewidth is present in the (18:0:1) sample, indicating relative immobility of the BF_4_ anions. In contrast, the (18:3:1) sample produces significantly sharper linewidths, consistent with a much higher percentage of mobile anions. ^23^Na NMR spectra would show the same trends across the different sample concentrations, with the (18:0:1) sample having the lowest cation mobility. Variable temperature investigations of the linewidth of the narrow component of the signals enabled the authors to estimate activation energies of about 42 kJ/mol for Na^+^ and BF_4_^−^ ions in the (18:0:1) sample, compared to 39 kJ/mol and 38 kJ/mol for Na^+^ and BF_4_^−^, respectively, in the 18:3:1 membrane. Despite the lack of measurable improvement in conductivity, the authors conclude that the succinonitrile has a significant effect on the ionic mobility in the membranes. In fact, the NMR lineshapes indicated multiple phases in the 18:3:1 sample—one immobile PEO:NaBF_4_ phase, and one more mobile PEO:SN:NaBF_4_ phase.

The system was further elucidated by ^13^C MAS NMR on the 18:3:1 system, which allowed the authors to estimate that about 1/3 of the PEO was in the mobile “SN-activated phase” after deconvolution of the broad and narrow PEO signal components. CPMAS measurements and J-coupling observed with the ^1^H decoupling deactivated would provide further evidence of this biphasic behavior. This is also not observed in similar Li-based membranes studied previously [113]. The presence of the immobile phase is thought to be the reason that the observed conductivity in the 18:3:1 membrane is less than that of the 18:0:1 sample, despite the significantly higher local ionic mobility. Pulsed-field gradient NMR may be useful in this case to establish a measurement to combine with the measured conductivity and reveal more information about the long-range dynamics of the system. However, due to the substantial nuclear quadrupole interactions resulting in rapid transverse relaxation associated with ^23^Na, PFG NMR would be a very challenging undertaking.

## 6. Conclusions

Nuclear magnetic resonance has proven to be an invaluable tool in the characterization of both structure and dynamics of a wide variety of materials. Its suitability for examining those properties associated with battery performance justifies its continued use in optimizing the electrolytes of the future; advances in both its technology and methodology allow it to remain relevant in the study of the ever-more complex electrolyte systems being developed. The quest for a practical room-temperature solid electrolyte continues, and the polymer electrolyte families described herein are but a small sample of the research towards viability.

## Figures and Tables

**Table 1:** Recent cell-level results reported from cells with dry solid polymer electrolytes. (Courtesy of Prof. Jay Whitacre, Carnegie Mellon University).

Dry Polymer Electrolyte Type	Room Temperature Conductivity Scm^−1^	Cathode Type Used	Cathode Loading Used (wt. % Active)	Areal Cathode Capacity	Test Fixture Format	Testing Temperature Used	# of Full/Deep Cycles Demonstrated
PEO/nanocomposite [21]	~10^−5^ or lower	LiFePO_4_ (<3.5 V)	60%	~1 mAh/cm^2^	Coin cell	100 °C	100
Polyether/LiFTSI [22]	~8 × 10^−5^	LiFePO_4_ (<3.5 V)	54%	Undisclosed	Coin cell	80 °C	1300
PEO/nano particle composite [23]	~5 × 10^−5^	LiFePO_4_ (<3.5 V)	63%	Undisclosed	Coin cell	70 °C	130
Single-ion BAB triblock copolymer [24]	Lower than 10^−6^	LiFePO_4_ (<3.5 V)	60%	8 mAh/cm^2^	Coin cell	80 °C	~100
Block Co-polymer (P3HT-PEO) [25]	~10^−5^ or lower	LiFePO_4_ (<3.5 V)	50%	Undisclosed	Coin cell	90 °C	10’s
Ordered Liquid Crystalline (meogen/Li salt) [26]	~10^−6^ Scm^−1^	LiFePO_4_ (<3.5 V)	65%	Undisclosed	Coin cell	60 °C	30
PEO/MEEGE [27]	~ lower than 10^−5^	LiFePO_4_ (<3.5 V)	83%	Undisclosed	Pouch Cell	60 °C	250
P(EO/MEEGE/AGE) [28]	lower than 10^−5^	Nano-coated LiCoO_2_	82%	~1 mAh/cm^2^	Coin cell	60 °C	25 (not fully stable at cathode potentials)
PEM [29]	<10^−3^	LiFePO_4_	80%	0.8–1.5 mg/cm^2^	Coin cell	ambient	50 cycles (80% capacity after)
Interlinked solid polymer electrolyte [30]	~10^−4^	LiFePO_4_ (2.5–4 V)	Undisclosed	~0.1 mAh/cm^2^	Coin cell	20 °C	50
Single ion triblock copolymer [31]	<10^−7^	LiFePO_4_	60%	Undisclosed	Undisclosed	70 °C	300 (77% capacity retention)
Carbonate-linked PEO electrolyte [32]	<10^−5^	LiFePO_4_	80%	1.3–1.8 mAh/cm^2^	Coin	25 °C	20
